# Cost-effectiveness analysis of ribotype-guided fecal microbiota transplantation in Chinese patients with severe *Clostridium difficile* infection

**DOI:** 10.1371/journal.pone.0201539

**Published:** 2018-07-26

**Authors:** Minghuan Jiang, Nok-hang Leung, Margaret Ip, Joyce H. S. You

**Affiliations:** 1 The Department of Pharmacy Administration and Clinical Pharmacy, School of Pharmacy, Xi’an Jiaotong University, Xi’an, Shaanxi, China; 2 The Center for Drug Safety and Policy Research, Xi’an Jiaotong University, Xi’an, Shaanxi, China; 3 The Global Health Institute, Xi’an Jiaotong University, Xi’an Jiaotong University, Xi’an, Shaanxi, China; 4 Shaanxi Center for Health Reform and Development Research, Xi’an, Shaanxi, China; 5 School of Pharmacy, Faculty of Medicine, The Chinese University of Hong Kong, Hong Kong SAR, China; 6 Department of Microbiology, Faculty of Medicine, The Chinese University of Hong Kong, Shatin, N.T, Hong Kong SAR, China; University Hospital Llandough, UNITED KINGDOM

## Abstract

**Background:**

*Clostridium difficile* infection (CDI) caused by ribotype 002 strain is associated with poor outcomes in Chinese patients. Fecal microbiota transplantation (FMT) is an effective but costly treatment for CDI. We aimed to examine potential cost-effectiveness of ribotype-guided FMT in Chinese patients with severe CDI.

**Methods:**

A decision-analytic model was designed to simulate outcomes of ribotype 002-guided FMT versus vancomycin treatment in Chinese patients with severe CDI in the hospital setting. Outcome measures included mortality rate; direct medical cost; and quality-adjusted life year (QALY) loss for CDI. Sensitivity analysis was performed to examine robustness of base-case results.

**Results:**

Comparing to vancomycin treatment, ribotype-guided FMT group reduced mortality (11.6% versus 17.1%), cost (USD8,807 versus USD9,790), and saved 0.472 QALYs in base-case analysis. One-way sensitivity analysis found the ribotype-guided FMT group to remain cost-effective when patient acceptance rate of FMT was >0.6% and ribotype 002 prevalence was >0.07%. In probabilistic sensitivity analysis, ribotype-guided FMT gained higher QALYs at 100% of simulations with mean QALY gain of 0.405 QALYs (95%CI: 0.400–0.410; p<0.001). The ribotype-guided group was less costly in 97.9% of time, and mean cost-saving was USA679 (95%CI: 670–688; p<0.001).

**Conclusions:**

In the present model, ribotype-guided FMT appears to be a potential option to save QALYs and cost when comparing with vancomycin. The cost-effectiveness of ribotype-guided FMT is subject to the patient acceptance to FMT and prevalence of ribotype 002.

## Background

*Clostridium difficile* infection (CDI) is the leading cause of diarrhea and pseudomembranous colitis with high 30-day mortality rate (15–25%) and substantial economic burden to the healthcare system [1]. Vancomycin and fidaxomicin are the recommended antibiotics for severe CDI [2]. Recurrent CDI occurs in 20–30% patients and increases risks of relapses, morbidity, and additional healthcare costs [3].

Fecal microbiota transplantation (FMT) is a procedure to restore the colonic fecal microbial diversity. Clinical findings supported FMT to be safe and effective for treatment of recurrent CDI, with significant higher cure rate than vancomycin [4–6]. The health economic outcomes of FMT for recurrent CDI was examined by decision analyses and the findings suggested FMT to be cost-effective over drug treatment (metronidazole, vancomycin, fidaxomicin) in the many regions [7–11]. Given the clinical efficacy and cost-effectiveness of FMT in patients with recurrent CDI, FMT is a potential treatment option for severe CDI. A retrospective cohort study on 3-month mortality of CDI reported early FMT to be an independent predictor associated with lower odds of mortality in severe CDI, but not in non-severe cases [12]. Nevertheless, serious adverse events including death were reported with the use of FMT [13], and the procedures of FMT preparation and administration are more costly than most drug therapy for CDI. To balance the effectiveness, safety and cost of FMT for treatment of severe CDI, predictors of poor outcome should be considered in the selection of patients for FMT treatment.

It has been reported that some strains of *C*. *difficle* were associated with poor treatment outcomes of CDI. The hyper-virulent strain ribotype 027 is correlated with enhanced disease severity and recurrence rate in the US and Europe [14,15]. An open prospective study reported that early FMT was associated with reduction of mortality in patients with ribotype 027 infection [16]. Ribotype 017 is increasingly found in Eastern European countries, South America and Asia, and it is also associated with recurrence and mortality [17,18]. In Hong Kong Chinese patients, the most commonly identified *C*. *difficile* strain is ribotype 002 [19,20]. An outcome study of CDI in Chinese patients found severe CDI and ribotype 002 were two independent predictors for death [19]. The *C*. *difficle* ribotype 002 is a potential predictor to select severe CDI cases for early FMT in Chinese patients. Ribotype-guided use of FMT is a possible approach to improve the clinical and economic outcomes of severe CDI. In the present study, we aimed to examine the potential cost-effectiveness of ribotype-guided FMT in Chinese patients with severe CDI from the perspective of healthcare provider.

## Methods

### Decision-analytic model

**Fig 1:**
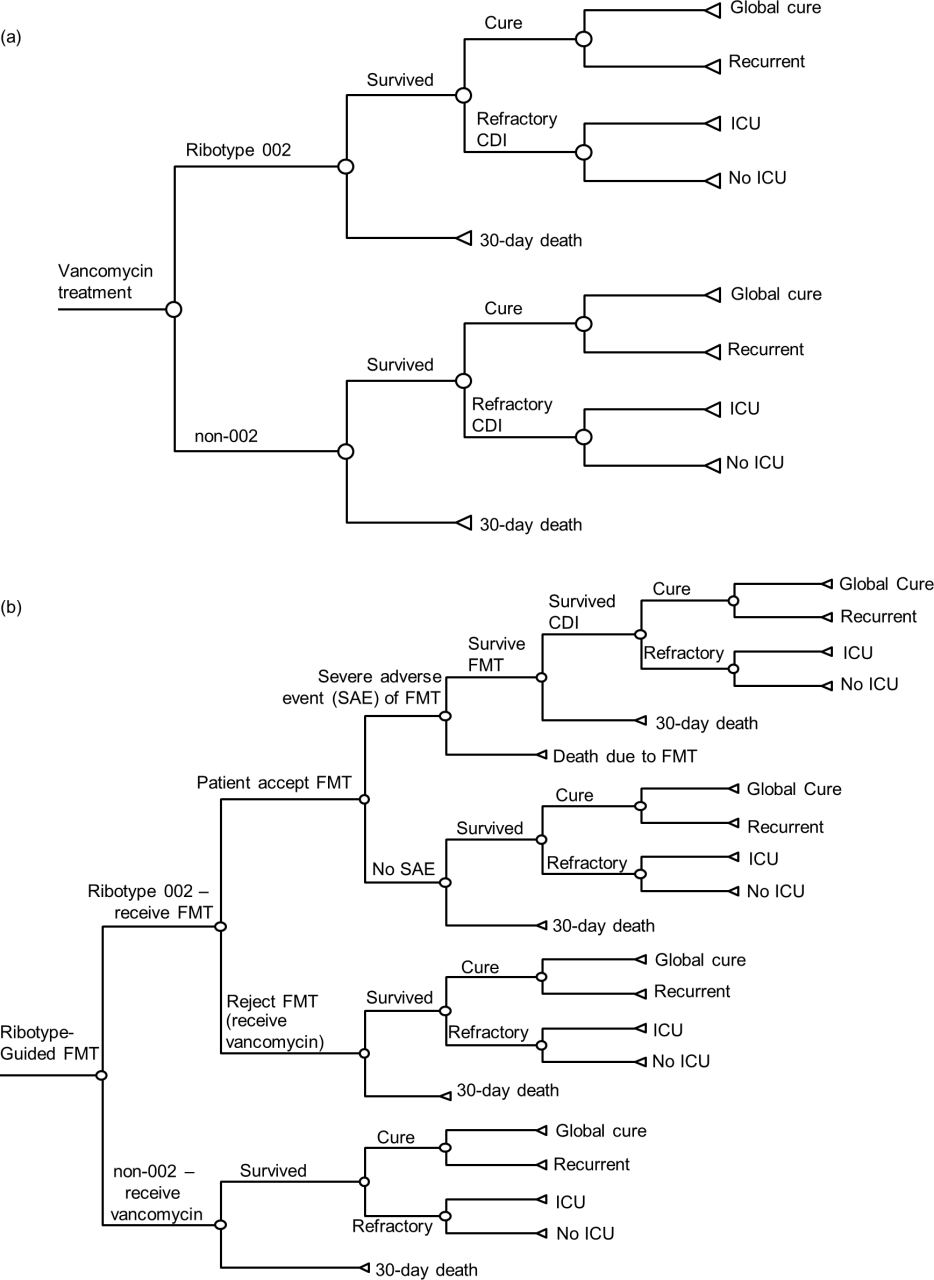
**Vancomycin treatment arm (a) and ribotype-guided FMT arm (b) of decision-analytic model for patients with severe CDI.** CDI: *Clostridium difficile* infection; FMT: fecal microbiota transplantation; ICU: intensive care unit.

A decision-analytic model was designed using TreeAge Pro 2016 software (TreeAge Software Inc., MA, USA) to simulate the outcomes of two therapeutic strategies in a hypothetical cohort of adult patients with severe CDI in the hospital setting. The two strategies were: Vancomycin 125mg four times per day by mouth (**Fig 1A**) and ribotype-guided FMT (**Fig 1B**). Inclusion criteria for model entry were adult patients with diagnosis of severe CDI. Severe CDI was defined by leukocytes >15000 cells/mL, or serum creatinine >1.5 mg/dL [2]. The time horizon of model was 30 days. The primary model outcomes included mortality rate; direct medical cost; and quality-adjusted life year (QALY) loss for CDI.

In the present model, patients in both arms were categorized by *C*. *difficile* ribotypes as 002 and non-002. In the vancomycin treatment arm, ribotyping was not performed and all patients received a standard course of oral vancomycin (125mg four times daily) [2]. In ribotype-guided FMT arm, *C*. *difficile* PCR-ribotyping was performed for all patients. The non-ribotype 002 cases were treated with the same regimen of vancomycin arm. Patients with ribotype 002 strain were treated with FMT, via lower gastrointestinal (GI) endoscopy, including oral vancomycin (500mg four times daily) 2 days before and 4 days after FMT [12]. Serious adverse events including death might occur to patients who received FMT.

In both study arms, a patient might experience clinical cure (resolution of diarrhea for the duration of therapy), and recurrent CDI might occur after cessation of initial therapy. By the end of model time horizon (30 days), a patient might be in one of the following health states: Global cure (resolution of diarrhea without recurrence), recurrent CDI (relapse of CDI after completion of CDI treatment), refractory CDI (diarrhea did not resolve after completion of CDI treatment) or death. Refractory cases might be admitted to intensive care unit (ICU). Recurrent and refractory CDI (not requiring ICU care) were subsequently treated with a course of FMT via lower endoscopy. Refractory cases at ICU care were treated as fulminant CDI with oral vancomycin and intravenous metronidazole [2].

### Clinical inputs

Literature search was conducted on Medline and Scopus over the period of 2000–2018 using keywords “ribotype 002”, “Chinese”, “*clostridium difficile* infection”, “severe *clostridium difficile* infection”, “recurrent *clostridium difficile* infection”, “vancomycin”, “fecal microbiota transplantation”, “adverse events”, “cure” and “mortality”. The selection criteria of clinical trials were: (1) Reports written in English; (2) patients in trials were aged 18 years or above; (3) prevalence of ribotype 002 in Chinese patients and association (if any) between ribotype 002 and treatment outcomes of CDI; (2) cure, recurrent, and mortality rates in 30 days with vancomycin or FMT for severe CDI; and (5) incidence of serious adverse events of FMT. Case reports were excluded. Preferred studies are meta-analyses or randomized controlled trials. If multiple sources were available for a model parameter, the weighted average was used as base-case value and the high/low values formed the range for sensitivity analysis.

Clinical parameters of the model were shown in **Table 1**. The prevalence of ribotype 002 (12.5%; range 9.4%-22.8%) in Chinese CDI patients were retrieved from a retrospective epidemiology study including 307 cases of CDI from five hospitals (one acute care and four chronic care) [20] and a prospective study in three Hong Kong acute-care hospitals on 100 cases of CDI [19].

**Table 1:** Model inputs.

Variables	Base-case value	Range	Distribution type	References
**Clinical inputs**				
Prevalence of ribotype 002	0.125	0.094–0.228	Beta	19,20
Mortality rate				
Ribotype 002	0.476	0.381–0.571	Beta	19
Non-ribotype 002	0.127	0.102–0.152	Beta	19
Vancomycin treatment				
Clinical cure rate	0.868	0.785–0.968	Beta	21–23
Recurrent rate	0.212	0.072–0.253	Beta	21–23
FMT via lower gastrointestinal endoscopy				
Clinical cure rate	0.930	0.800–1.000	Triangular	25–27
Odds ratio of mortality with early FMT	0.075	0.016–0.34	Triangular	12
Incidence of serious adverse event with FMT	0.061	0.0488–0.0732	Beta	13
Mortality rate among serious adverse events of FMT	0.025	0.02–0.03	Beta	13
CDI-related intensive care unit admission in refractory cases	0.099	0.079–0.119	Beta	24
Duration (days)				
Attributable CDI length of hospitalization	7	5–9	Uniform	24,30
Vancomycin treatment course	12	10–14	Uniform	2,31
Length of hospitalization for serious adverse event of FMT	7	5–10		Assumption
**Utility inputs**				
Age of patients with initial CDI	72	58–88	Triangular	19
Healthy adults aged 58–65 years	0.92	-	-	28
Healthy elderly aged 66–88 years	0.84	-	-	28
Initial CDI	0.82	0.72–0.84	Triangular	7
Recurrent CDI	0.82	0.72–0.84	Triangular	7
Refractory CDI	0.71	0.5–0.72	Triangular	7
Disutility of intensive care unit	-0.34	-(0.27–0.41)	Triangular	29
Serious adverse event of FMT	-0.34	-(0.27–0.41)	Triangular	Assumption
**Cost inputs (USD)**				
Daily cost of vancomycin (125mg four times daily by mouth)	2.9	2.3–3.6	Uniform	Local cost
Daily cost of antibiotic treatment for fulminant CDI				
Vancomycin 500mg four times daily by mouth	12	9–14	Uniform	Local cost
Metronidazole 500mg every 8 hours intravenously	1.2	0.9–1.4	Uniform	Local cost
Daily cost of general medical ward	654	-	-	Local cost
Daily cost of intensive care unit	3,128	-	-	Local cost
Cost of *Clostridium difficile* toxin	26	21–32	Uniform	Local cost
Cost of ribotyping test	128	103–154	Uniform	Local cost
Cost of management for serious infection of FMT	8,305	1,886–62,992	Triangular	33
Number of FMT received	1	1–3	Triangular	12
Cost of FMT				
Donor tests	525	420–630	Uniform	Local cost
Recipient tests	118	94–142	Uniform	Local cost
FMT preparation	65	52–78	Uniform	Local cost
Bowel preparation	12	10–14	Uniform	Local cost
Vancomycin before and after FMT	18	14–22	Uniform	Local cost
Lower gastrointestinal endoscopy	640	512–768	Uniform	Local cost

CDI: *Clostridium difficile* infection; FMT: fecal microbiota transplantation

The clinical cure rate of vancomycin for severe CDI (86.8%; range: 78.5%-96.8%) and incidence of recurrent CDI (21.2%; range: 7.2%-25.3%) were estimated from the findings of three prospective clinical trials (total 646 cases treated with vancomycin) [21–23]. In a prospective case-control outcome study of CDI (139 cases and 114 controls), the mortality in ribotype 002 cases (47.6%) was higher than those with other ribotypes (12.7%) and ribotype 002 was found to be an independent predictor for mortality (HR 2.8, 95% CI 1.1–7.2; p = 0.03) [19]. The mortality rates of ribotype 002 and non-ribotype 002 cases were adopted by the present model. Ribotype 002 was not identified to associate with refractory or recurrence, and we therefore applied the same cure and recurrent rates to both ribotype 002 and non-ribotype 002 cases. The CDI-related ICU admission rate (9.9%) for refractory cases was retrieved from a retrospective case-control outcome study on events attributable to CDI in hospitalized patients (161 cases and 656 control) [24].

The cure rate of FMT via lower GI endoscopy (93%; range: 80%-100%) was estimated from data reported in two systematic reviews and an comparative study on effectiveness of FMT by route of administration [25–27]. The odds ratio of 3-month mortality with early FMT (via lower GI route) versus antibiotic treatment (0.075; 95%CI 0.016–0.34; p = 0.001) was reported in a retrospective outcome study of severe CDI patients (n = 111) [12]. The serious adverse event rate of FMT via lower GI routes (6.1%; range: 4.9%-7.3%) and the mortality rate among serious events (2.5%; range: 2%-3%) were retrieved from a systematic review including 50 publications on adverse events of FMT [13].

### Utility inputs

The QALY loss for CDI-associated health states (CDI, recurrent CDI, refractory CDI, serious adverse event of FMT if occurred) in each patient was estimated by loss of utility and the patient-time spent in each state. The loss of utility was approximated by age-specific utility for healthy individual minus health state-specific utility. The base-case value of patient age in the present model was 72 year (range 58–88 years), adopted from the mean age of 139 CDI patients in a local prospective outcome study [19]. Age-specific utility scores of healthy individuals aged 18–64 years (0.92) and aged ≥65years (0.84) were retrieved from a US national health-related quality of life study [28]. Disease-specific utility values for CDI, recurrent CDI and refractory CDI were adopted from the model input values of a cost-effectiveness analysis on four management strategies for CDI treatment [7]. The utility value for refractory cases managed in ICU was further lower by disutility of ICU care (-0.34) [29]. Serious adverse events of FMT via lower GI routes included a broad spectrum of 44 events [13]. Whilst the clinical model input for serious adverse event rate was an estimated value including a variety of events, severe infection requiring ICU care was used as the index event for estimation of QALY loss and cost of serious adverse event in our model. Duration of CDI illness (as time-spent for antibiotic treatment and FMT treatment) were estimated from clinical trials and treatment guidelines [2,12,24,30,31]. The length of treatment for serious adverse event of FMT was assumed to be 7 days (range 5–10 days). The QALYs loss from death was calculated by using age-specific utility score and time loss for death. Time loss for death was retrieved from projected age-specific life expectancy reported by Hong Kong Census and Statistics Department [32]. QALYs loss for death was discounted to year 2018 with an annual rate of 3%.

### Cost inputs

The cost analysis was conducted from the perspective of healthcare provider in Hong Kong using direct medical costs at year 2018. Cost of FMT (from universal stool bank) was estimated using local pricing, including FMT preparation, bowel preparation, 6-day vancomycin pre- and post-FMT, donor and recipient testing. The attributable CDI length of hospitalization was estimated from outcome analyses of CDI in the hospital setting [24,30]. The cost of ribotyping test was estimated from a microbiology laboratory in a public hospital of Hong Kong. The management cost of serious infection (as index serious adverse event of FMT) was derived from a health economic study in Hong Kong [33].

### Cost-effectiveness analysis, sensitivity analysis, and scenario analysis

Expected mortality, direct medical cost and QALY loss were calculated for each study arm in base-case analysis. When ribotype-guided FMT gained higher QALYs at additional cost, the incremental cost-effectiveness ratio (ICER) was calculated using the following equation: (Cost _ribotype-guided FMT_—Cost _vancomycin treatment_)/(QALY loss _vancomycin treatment_—QALY loss _ribotype-guided FMT_). The World Health Organization suggested a strategy to be considered as highly cost-effective if the ICER was lower than 1× gross domestic product (GDP) per capita of the jurisdiction [34]. The GDP per capita in Hong Kong was USD43,530 (USD1 = HKD7.8) in 2016, and was adopted as the willingness-to-pay (WTP) threshold [35]. Ribotype-guided FMT was considered as the preferred option if it saved QALYs at lower cost, or it saved QALYs at higher cost with ICER lower than 43,530 USD/QALY.

Sensitivity analysis was performed by TreeAge Pro 2016 software (TreeAge Software Inc., MA, USA) and Microsoft Excel 2016 (Microsoft Corporation, WA, USA) to examine the robustness of base-case results. One-way sensitivity analysis was conducted on all model inputs over the ranges listed in **Table 1**. To evaluate the impact of uncertainty of all variables simultaneously, probabilistic sensitivity analysis was performed using Monte Carlo simulation. Direct cost and QALY loss of both study arms were recalculated 10,000 times by randomly drawing each model input from the probability distribution indicated in **Table 1**.

In base-case analysis, we assumed 100% patients with ribotype 002 in the ribotype-guided FMT arm to accept FMT treatment. A scenario analysis was performed to examine the impact of acceptance rate of FMT treatment (over wide range of 0%-100%) and to identify the threshold acceptance rate for ribotype-guided FMT to be cost-effective.

## Results

### Base-case analysis

The results of base-case analysis were shown in **Table 2**. Comparing with vancomycin treatment, the ribotype-guided FMT arm reduced mortality by 32.2% (11.6% versus 17.1%), saved 0.472 QALYs and reduced cost per patient by 10.0% (USD8,807 versus USD9,790). Ribotype-guided FMT was therefore the preferred option in the base-case analysis. The number needed to treat to prevent one death was 18.

**Table 2:** Base-case results of expected mortality, cost, and quality-adjusted life-year (QALY) loss.

Strategy	Mortality	Cost (USD)	QALY loss
Ribotype-guided FMT	0.116	8,807	0.998
Standard treatment	0.171	9,790	1.470

CDI: *Clostridium difficile* infection; QALY: quality-adjusted life-years

### Sensitivity analysis and scenario analysis

**Fig 2:**
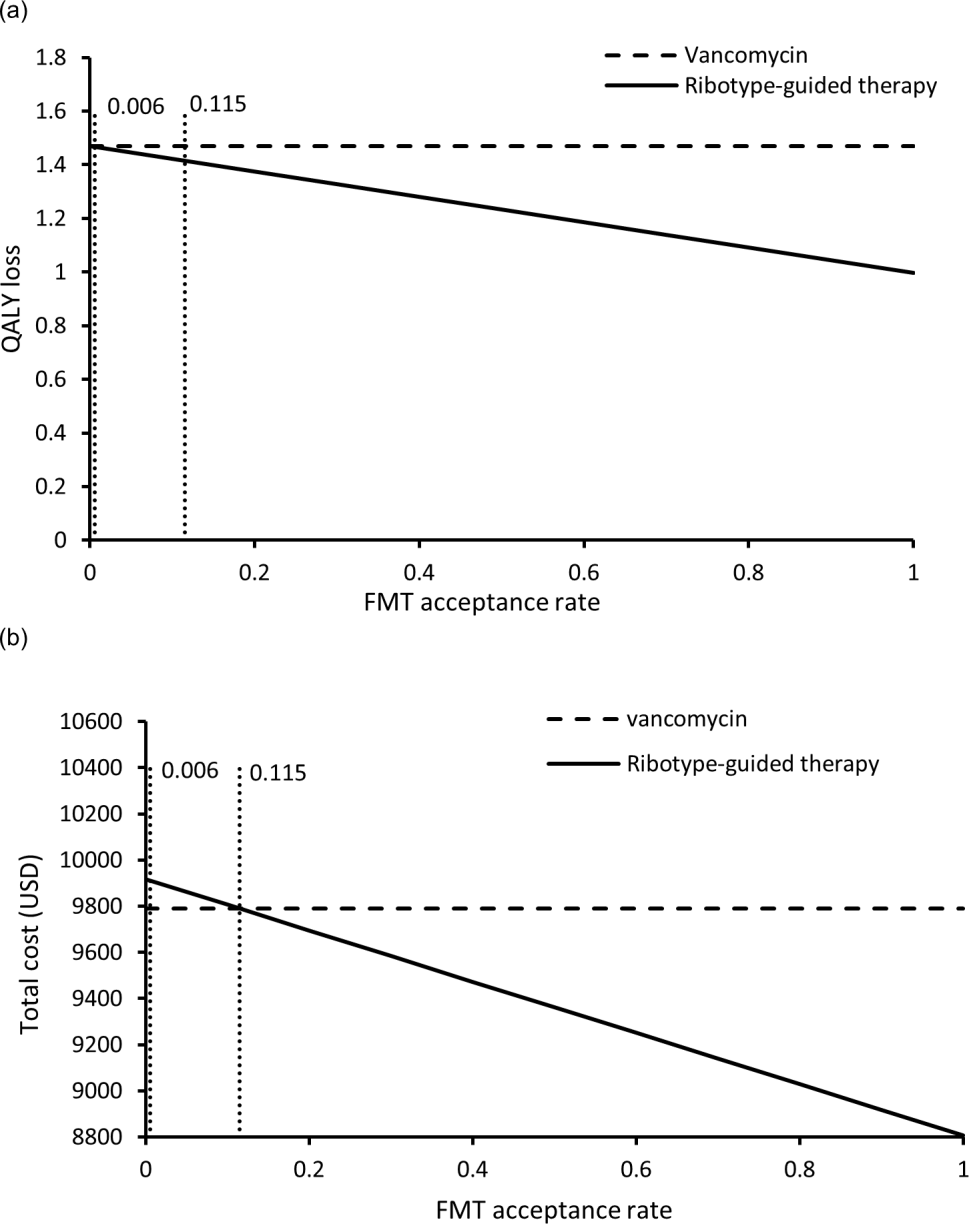
QALYs loss (a) and total cost (b) of each strategy against variation of patient acceptance rate to FMT.

One-way sensitivity analysis found the base-case results to be robust and no threshold value was identified throughout variation of all model parameters. The results of scenario analysis showed that ribotype-guided FMT remained to save QALYs throughout the variation of patient acceptance to FMT (from >0% to 100%) (**Fig 2A**). The QALY loss in both arms were identical when the patient acceptance was 0%. Ribotype-guided FMT arm was less costly than vancomycin group when patient acceptance exceeded 11.5% (**Fig 2B**). When patient acceptance varied between 0.6%-11.5%, the ribotype-guided FMT saved QALYs at higher cost with ICER below WTP threshold (43,497 USD/QALY) and was therefore the preferred option. When the patient acceptance rate was lower than 0.6%, the ICER of ribotype-guided FMT exceeded the WTP threshold and vancomycin treatment became the preferred option.

**Fig 3:**
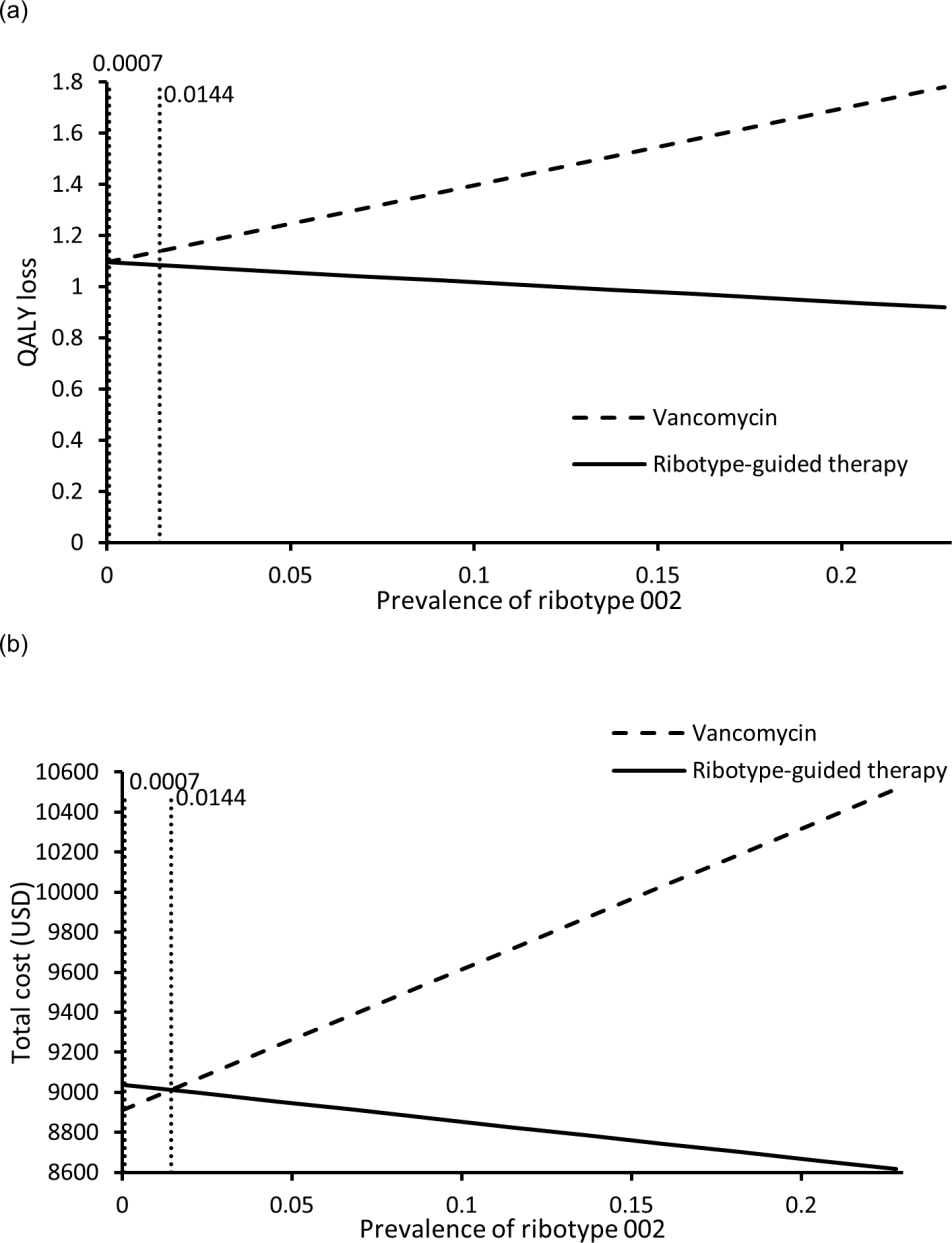
QALYs loss (a) and total cost (b) of each strategy against variation of prevalence of ribotype 002.

To examine the impact of ribotype 002 testing on the cost-effectiveness analysis, one-way sensitivity analysis was further conducted with extended ranges of ribotype 002 prevalence (0%-100%) and ribotyping cost (USD103-USD500). Ribotype-guided FMT saved QALYs when ribotype 002 prevalence ranged between >0% to 100% (**Fig 3A**), and it became more costly than vancomycin treatment at prevalence <1.44% (**Fig 3B**). The ICER of ribotype-guide FMT was lower than the WTP threshold at ribotype 002 prevalence 0.07%-1.44%, and it exceeded the WTP threshold when the prevalence was lower than 0.07%. Ribotype-guided FMT remained to be cost-saving over the extended range of ribotype cost.

**Fig 4:**
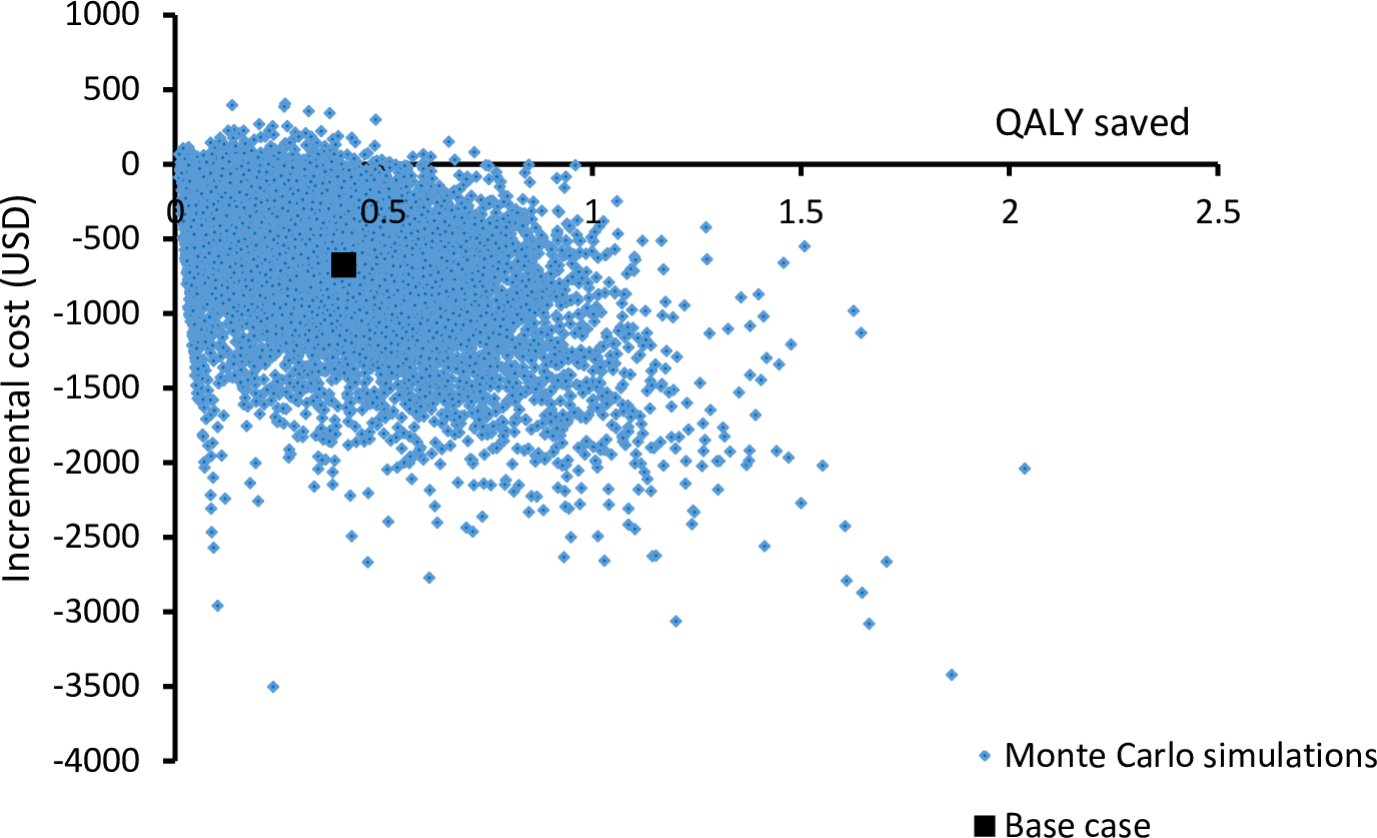
Scatter plot of incremental cost against incremental QALYs of ribotype-guided FMT versus vancomycin treatment.

Probabilistic sensitivity analysis was performed by 10,000 Monte Carlo simulations. **Fig 4**showed a scattered plot of incremental total cost versus QALYs saved by ribotype-guided FMT comparing to vancomycin treatment. Ribotype-guided FMT gained higher QALYs in 100% of simulations and the mean QALYs saved was 0.405 QALYs (95%CI: 0.400–0.410; p<0.001). The ribotype-guided group was cost-saving in 97.9% of time. In 2.1% simulations, the ribotype-guided FMT group saved QALYs at higher cost, with ICERs (median 261 USD/QALY; range: 0.5–6,286 USD/QALY) below the WTP threshold. The mean cost saving in 10,000 simulations was USD679 (95%CI: 670–688; p<0.001).

## Discussion

This is the first cost-effectiveness analysis of ribotype-guided use of FMT for patients with severe CDI. Base-case analysis found ribotype-guided FMT to be the preferred strategy with QALYs saved at lower cost when compared to vancomycin therapy. The findings of probabilistic sensitivity analysis supported ribotype-guided FMT to be the preferred option in 100% of 10,000 Monte Carlo simulations (97.9% simulations were effective and cost-saving and 2.1% were cost-effective with ICER< WTP threshold (USD 43,497 USD/QALY)).

A previous study examining patients’ attitude on FMT reported that FMT was not appealing in 75% patients [36]. The patient acceptance to FMT could potentially reduce the cost-effectiveness of ribotype-guided FMT treatment approach. In base-case scenario, we assumed 100% patients with ribotype 002 to accept FMT, and ribotype-guided FMT was more effective at lower cost compared to vancomycin treatment. In the scenario analysis, the acceptance of FMT was examined over 0%-100%. The effectiveness of ribotype-guided FMT was highly robust that it saved QALYs if the acceptance to FMT was above 0%. The cost-saving of ribotype-guided FMT reduced when the acceptance rate declined, yet it remained highly cost-effective (ICER <1xGDP per capita of Hong Kong) between acceptance rate of 0.6%-11.5%. At acceptance rate less than 0.6%, nearly all patients in the ribotype-guided arm received vancomycin treatment. The QALYs saved by FMT in few cases did not offset the cost of ribotyping test in all patients.

The impact of ribotyping cost on the base-case results was further examined with higher upper limit (USD500) and no threshold value was identified. Extended sensitivity analysis on prevalence of ribotype 002 found that the ribotype-guided FMT remained effective and cost-saving at ribotype 002 prevalence above 1.44%. At extremely low prevalence of ribotype 002 (0.07%-1.44%), the ribotype-guided FMT was effective at higher cost, yet still accepted as cost-effective per WTP threshold.

A previous cost-effectiveness study examined the cost and QALYs of universal FMT versus vancomycin in patients with initial CDI (including both severe and non-severe CDIs) from the perspective of US healthcare payer [37]. Donor stool was administered to all patients in FMT-treated arm. The reported findings suggested that universal FMT was less costly than universal vancomycin for initial CDI by USD221. The cost-saving was mostly generated from the lower cost input of FMT treatment (USD1086) versus vancomycin treatment (USD1347) in this prior analysis. In Hong Kong, the drug treatment with oral vancomycin was much lower, and the total cost input (USD1378) of FMT via lower GI route was nearly 40-fold higher than the cost input (USD2.9 per day for 12 days: USD35) of oral vancomycin treatment course in our analysis. In the present model, we included ribotype 002 for selection of severe CDI cases with high risk for mortality to receive FMT in Chinese patients. Our findings were consistent with the previously reported study that the FMT group had higher gain in QALYs. By ribotype-guided selection of high-risk cases, both cost and QALY saving were balanced and cost-effective use of FMT was achieved in the present model.

CDI is a major burden to the healthcare system with extended hospitalization and rehabilitation [38]. FMT is recommended as an option for those who failed standard treatment, especially patients with recurrent CDI. Due to the less appealing procedure, patients’ attitude and acceptance might influence the selection of treatment regimen. The ribotyping results might assist patients in the process of informed decision-making on acceptance of FMT. Selecting severe CDI cases with high-risk ribotype (demonstrated with strong association to severe outcomes such as ICU admission and death) for FMT is a potential approach in CDI management. The results of present study provided insights on application of *C*. *difficile* ribotype information for cost-effective use of FMT in patients with severe CDI. Ribotype 002 is the *C*. *difficile* strain associated with poor outcomes in Hong Kong Chinese patients, whilst the association between ribotype 027 (the prominent strain with major outbreaks and serious infections in North America and Europe) and severe outcomes was controversial [39]. Despite that, the model frame-work developed in the present study is readily to be adopted by healthcare systems in other parts of the world, using system-specific practice and costs of healthcare resources, region-specific high-risk ribotype and the corresponding prevalence. Our findings supported prospective randomized clinical trials to examine the outcomes of applying *C*. *difficile* ribotype 002 information to guide the selection of severe CDI cases to offer FMT treatment. Further studies to investigate the efficacy, safety and cost-effectiveness of FMT for treatment of all cases of severe CDI in Chinese patients are also warranted to generate important data to inform clinical practice as well as decision-analytic models for robust translation to cost-effective treatment approach.

There are several limitations in the present study. Model-based analyses are generally subject to the uncertainty of model inputs. International pharmacoeconomic guidelines on data transferability consider relative treatment effect to have high transferability even if derived from clinical trials conducted in a population different from the local population. Baseline prevalence is considered to have low transferability and should be sourced from jurisdiction of interest or similar locations. Healthcare resources utilization and unit costs of healthcare resources are also considered to have low transferability, and local data and pricing should be used [40]. The clinical inputs of FMT were retrieved from studies reported in overseas due to limited data available in Chinese patients. Many of the patients in these studies were treated for recurrent CDI, and we assumed the clinical cure rate of FMT in patients with severe CDI was similar to patients with recurrent CDI. The uncertainty in clinical inputs of FMT might over or under estimate the benefits of FMT in the present hypothetical cohort. Rigorous sensitivity analyses was therefore performed to examine the impact of uncertainty of all variables on the robustness of model outcomes. The turn-around-time of ribotyping test is a critical factor on the feasibility of adopting ribotyping into the treatment strategy in clinical trials. The approximated laboratory run time of *C*. *difficile* ribotyping test was reported to be 6.8 hours [20,41], yet the turn-around-time of ribotyping test was subject to the operational and administration procedures of microbiology laboratory in different hospital settings. The cost of ribotyping in Hong Kong was estimated to be USD128. Introduction of improved and low-cost (<USD10) technology of ribotyping [42] to Hong Kong could reduce the testing cost and further increase the cost-saving of ribotype-guided treatment. The present model assumed the stool are readily available in stool bank from healthy volunteers. In the healthcare setting without access to stool bank, FMT using patient directed donor is more time- and cost-consuming and might therefore affect the cost-effectiveness of FMT. Fidaxomicin is another recommended treatment option for severe CDI, yet it is not marketed in Hong Kong. The present model therefore did not included fidaxomicin. The cost analysis was conducted with direct medical cost from the perspective of healthcare provider. Indirect cost (loss of productivity in patients and care-takers) was not included, and the cost analysis might therefore underestimate the economic benefits of ribotype-guided FMT.

## Conclusions

In the present model, ribotype-guided FMT appears to be a potential option to save QALYs and cost when comparing with vancomycin. The cost-effectiveness of ribotype-guided FMT is subject to the patient acceptance to FMT and prevalence of ribotype 002.
